# Interaction between *Pax6* and its novel mutant in *Bufo raddei* Strauch

**Published:** 2011-10-18

**Authors:** Furong Ju, Yongqing Zhao, Yuanlin Zhao, Ying Wang, Fan Wen, Lin Ye, Lan Gao

**Affiliations:** School of Life Science, Lanzhou University, Gansu, China

## Abstract

**Purpose:**

Exploration of the relationship between a novel paired box 6 (*Pax6*) mutant and *Pax6* in *Bufo raddei* Strauch.

**Methods:**

RT–PCR, yeast 2-hybrid system, and co-immunoprecipitation were used to analyze the Pax6 protein and its mutant during embryonic eye development in *Bufo raddei* Strauch.

**Results:**

We have cloned the *Pax6* ORF sequence from *Bufo raddei* Strauch. Here we report the cloning of a novel *Pax6* homolog of *Bufo raddei* Strauch named *Pax6* variant. Comparing the 2 genes, the homolog of ORF nucleotide sequence is more than 99% in *Bufo raddei* Strauch; only the proline-serine-threonine(PST)-rich transaction domain differs. The deduced amino acid sequences of PST region are 53.1% identical. An interaction was found between *Pax6* and *Pax6* variant via yeast 2-hybrid system; with further study, we found that they interacted in vivo via co-immunopricipitation.

**Conclusions:**

A Pax6 mutant was first found in *Bufo raddei* Strauch. Interaction between Pax6 and Pax6 variant may play a critical role during eye development in *Bufo raddei* Strauch. This suggests that expression of *Pax6* variant may play a role and appears to be a necessity in eye development, but that *Pax6* itself is still pivotal in eye development.

## Introduction

Paired box 6 (*Pax6*) is expressed throughout eye development [1-3], and has been reported to be a master of eye development. However, in true moles (*Talpidae*), Pax6 was found to be dispensable [4], although it was recently found to be pivotal in initiation of lens fiber cell differentiation [5]. Pax6 is also important in the central nervous system. Via alpha-crystallin, Pax6 regulates survival of dopaminergic olfactory bulb neurons [6]. In the forebrain, accurate expression of *Pax6* is important for cortical progenitor proliferation [7]; in the rat hindbrain, Pax6 is required for coordinating boundary-cell specification, and within the hindbrain, boundary region neurogenesis can be reduced [8]. In the postnatal limbic system, *Pax6* plays a key role in the generation of multiple subtypes of neurons [9]. In postnatal cerebellum development, *Pax6* is pivotal in the life of the developing granule cell [10], and by an essential cephalic ectodermal patterning center, Pax6 can regulate craniofacial form [11]. *Pax6* is crucial for cell development; it plays an essential role in controlling the expression of the key genes involved in pancreatic alpha cell differentiation and function [12]. In mouse embryonic stem cells, Pax6 can induce retinal neuron progenitors [13], and the epidermal growth factor-responsive neural stem cells can be regulated by Pax6 [14]. Overexpression of *Pax6* can change development and function in some cells, and the level of Pax6 directly maintains normal corneal epithelial cells [15], but inhibits the growth of cultured human retinoblastoma cells [16]. Via the MET tyrosine kinase receptor, Pax6 participates in cancer progression [17]; some researchers have found that glioma angiogenesis can be restrained by Pax6 [18]. *Pax6* expression levels can be controlled by forkhead box G1 (*Foxg1*) in telencephalic progenitor proliferation cells [19]. Some genes, like sine oculis homeobox 3 (*Six3*) and orthodenticle homeobox (*Otx*), may play a prominent role in *Pax6* expression in the early development of the stalked crinoid Metacrinus rotundus [20]. It was reported that *Pax6* was connected with hormones; Pax6 can restrain androgen receptors by reducing coactivators to AR target promoters [21], and can influence islet function via its variant, which is shown to reduce *Pax6* expression in human islets [22]. In the endocrine pancreas, some transcription factors are involved in the activation of the glucagon gene, which is controlled by Pax6 [23].

Pax6 includes 3 functional domains: the paired box, the paired homeobox, and the proline, serine, threonine–rich (PST-rich) region [24]. Some Pax6 mutations have only partial homeodomain or none at all [25,26]. The loss of 1 functional allele of *Pax6* leads to mouse small eye [27,28], Peter’s anomaly, and congenital cataracts in human eye development [29,30]. Some novel mutations in *Pax6* can cause the classic aniridia phenotype [31]. In disease research, some *Pax6* mutant rats serve as a model for autism [32].

*Pax6* homolog has now been described in other invertebrates, such as flatworm, ribbon worm, *C. elegans*, squid, sea urchin, and ascidian [33]. Some species also contain a second DNA-binding domain, the paired-type homeodomain that is separated from the NH_2_-terminal [34,35]. The COOH-terminal domain is not conserved between vertebrates, *Drosophila* eyeless, and the ascidian Pax6 protein, but they may have biochemical functions [36-39]. Most mutations of *Pax6* result in specific defects in the development of the eye; some can influence the nuclear signal transfer, and others can influence the combination of the DNA match domain [26]. Through direct or indirect protein–protein interaction, the efficiency of transcription factor can be improved [40]. *Pax6* mutations lead to several ocular defects; with *Pax6*-consensus binding sequence, *Pax6*(5a)-consensus binding sequence, and homeodomain binding sequence containing luciferase reporters, the transactivation potential of *Pax6*(5a)-G36A was enhanced [41].

We conducted a study in amphibians, focusing on *Bufo raddei* strauch, which is distributed in the northern part of China, and is even found in saline-alkali soil, given its strong adaptability. It serves as the consumer lying at the base of the food chain; it is an important species in maintaining the diversity of species and the stability of the ecosystem. This species is gonochorism and ovulate in water and then the eggs grow into tadpoles. In winter, when the temperature falls below 10 °C, they initiate hibernation in caves. *Bufo raddei* strauch belongs to anura bufonidae. The optic vesicle can be clearly observed when the embryon of *Bufo raddei* strauch reach 6 days; after 7.5 days, the pigment of cornea begins to reduce and the lens begins to appear.

## Methods

### Animal

*Bufo raddei* Strauch embryos were obtained at 6 days’ growth.

### Gene clones and protein expression

Total RNA was isolated from the *Bufo raddei* Strauch when the embryos grew to 6 days, and then was reverse transcribed into cDNA. The sequences of the entire open reading frame for the full-length *Bufo raddei* Strauch *Pax6* and *Pax6* variant was amplified by means of polymerase chain reaction; at the same time, vectors were successfully constructed (Table 1).

**Table 1:** Primers of plasmid which was constructed for protein purification.

**Plasmid**	**Primers**	**Vector**	**Enzyme cut sites**	**Fragment size**
pET-28-Pax-6	5′-CCGGAATTCATGCAGAACAGTCACAGCGGA-3	pET28	EcoR I, Hind III	1270
	5′-CCCAAGCTTCTGTAGTCTTGGCCAGTACTG-3′			
pET-28-Pax-6 variant	5′-CCGGAATTCATGCAGAACAGTCACAGCGGA-3	pET28	EcoR I, Hind III	1182
	5′-AAAAAGCTTTAAAATACTGCTGAACATCC-3′			
pET-28-Gfp-Pax-6	5′-AAACCATGGGTAAAGGAGAAGAAC-3	pET28	NcoI, Hind III	948
	5′GTCTGGCTGGGTACAGGGGGTTTGTATAGTTCATCCATG-3′			
	5′CATGGATGAACTATACAAACCCCCTGTACCCAGCCAGAC-3′			
	5′TTTAAGCTTCTGTAGTCTTGGCCAGTACTG-3′			
pET-28-Gfp-Pax-6-variant	5′-AAACCATGGGTAAAGGAGAAGAAC-3′	pET28	NcoI, Hind III	864
	5′-CTCCAGGGGAAATGAGACTTTGTATAGTTCATCCATG-3′			
	5′-CATGGATGAACTATACAAAGTCTCATTTCCCCTGGAG-3′			
	5′-AAAAAGCTTTAAAATACTGCTGAACATCC-3			

The Table displays the specific primers of each gene, and appropriate vector, enzyme cut sites and product length.

The plasmid for pET-28-Gfp-Pax6 included a partial sequence of *Pax6* ORF, only 231 bp in the PST region, which has 432 bp. The pET-28-Gfp-Pax6-variant has only 147 bp in PST region, which has 348 bp of *Pax6* variant ORF sequence. All recombinants were verified by DNA sequencing. Four proteins were expressed in Rosetta (DE3) cells and purified via nickel affinity column; these were Pax6-His, Pax6-variant-His protein, Gfp-Pax6-His, and Gfp-Pax6-variant-His.

### Preparation of antiserum against Gfp-Pax6 and Gfp-Pax6 variant

Per Bradford, the concentration of purified fusion proteins was examined. First, 1 mg of Gfp-Pax6 protein was mixed with 1 ml Freund's incomplete adjuvant (Invitrogen, Shanghai, China), and then injected into a rabbit. After 3 days, 1 ml Freund's incomplete adjuvant (Invitrogen) was replaced by 1 ml Freund's complete adjuvant, which was mixed with antigen protein, and was injected into the rabbit. The same operation was conducted 3 times at weekly intervals. At the same time, Gfp-Pax6-variant protein replaced the Gfp-Pax6 protein, and was injected into another rabbit using the same method. Finally, polyclonal antisera were obtained and purified.

### Yeast two-hybrid system

Four recombinants were obtained by primers, as shown in Table 2. The entire open reading frame of *Pax6* and *Pax6* variant was cloned and digested by Nde1 and EcoR1 (Takara, Tokyo, Japan). They were respectively linked to pGBK-T7 vectors which were digested by the same enzymes. Meanwhile, *Pax6* and *Pax6* variant were digested by EcoR1 and Xho1, respectively, and linked to pGAD-T7 vectors, which were digested by the same enzymes. These 4 recombinants were verified by DNA sequencing, and were transferred into Yeast Y190. Six experimental groups were constructed: Pax6-pGAD-T7 and Pax6-variant-pGBK-T7, Pax6-pGBK-T7 and Pax6-variant-pGAD-T7, Pax6-pGAD-T7 and pGBK-T7, Pax6-variant-pGBK-T7 and pGAD-T7, Pax6-pGBK-T7 and pGAD-T7, and Pax6-variant-pGAD-T7 and pGBK-T7. Each group was transferred into Yeast Y190, so that 6 different yeast expression systems were produced. After they grew in culture medium, which lacked Leu and Trp, for 3 days, the surviving yeast colony was transferred to a culture medium containing dicyandiamide and that lacked Leu, Trp, and His, where some of the yeast colony still continued to grow. The surviving yeast colony was transferred into nitrocellulose membrane, frozen by liquid nitrogen for 30 s, then melted at room temperature. Then the membrane carrying some yeast colony was incubated in buffer (60 mM Na_2_HPO_4_, 40 mM NaH_2_PO_4_, 1 mM KCl, 5 mM MgSO_4_, 0.27% (v/v) β-mercaptoethanol, 35mg/ml X-gal) to observe color change.

**Table 2:** Primers of plasmid which was constructed for yeast two-hybrid system.

**Plasmid**	**Primers**	**Vectors**	**Enzyme cut sites**	**Fragment size**
Pax-6- pGBK-T7	5′-GGAATTCCATATGATGCAGAACAGTCACAGCGG-3′	pGBK-T7	Nde1, EcoR1	1288
	5′-CCGGAATTCCTGTAGTCTTGGCCAGTACTG-3′			
Pax-6-pGAD-T7	5′-CCGGAATTCATGCAGAACAGTCACAGCGG-3′	pGAD-T7	EcoR1, Xho1	1285
	5′-CCGCTCGAGCCTGTAGTCTTGGCCAGTACTG-3′			
Pax-6-variant-pGAD-T7	5′-CCGGAATTCATGCAGAACAGTCACAGCGG-3′	pGAD-T7	EcoR1, Xho1	1198
	5′-CCGCTCGAGCTAAAATACTGCTGAACATCC-3′			
Pax-6-variant- pGBK-T7	5′-GGAATTCCATATGATGCAGAACAGTCACAGCGG-3′	pGBK-T7	Nde1, EcoR1	1201
	5′-CCGGAATTCTAAAATACTGCTGAACATCC-3′			

The Table displays the specific primers of each gene, and appropriate vector, enzyme cut sites and product length.

### Co-immunoprecipitation (Co-IP)

The embryos were collected at 6 days after fertilization. The samples were pooled and ground to a powder with liquid nitrogen. The powder was dissolved in lysis buffer that contained 100 mM Na_2_HPO_4_, 1 mM EDTA, 1 mM EGTA, 5% (v/v) glycerol, 5 mM MgCl_2_, 100 mM KCl, 10 mM NaCl, 0.1% (v/v) Triton X-100 (Invitrogen), 0.1% (V/V) Tween-20 (Invitrogen), 10 mM Tris-HCl, 35μg/ml PMSF (Invitrogen), 0.5μg/ml Leupeptin (Invitrogen) pH 7.5) [42,43]. After incubation on ice for 30 min, it was centrifuged at 13,000× g at 4 °C for 30 min. The supernatant was collected and we proceeded with determination of protein concentration. Then, 1 mg total protein was incubated with 1 μg rabbit anti-gfp-Pax6 antibody in a total volume of 1 ml. We use anti-GST antibody in a negative control group. After 2 h incubation at 4 °C, the protein and antibody mix was incubated with 20 μl of magnetic Protein A (Sigma-Aldrich, Shanghai, China) particles at 4 °C for 1 night with shaking. Particles were separated with a magnetic stand and then washed 4 times with wash buffer: TBST. Particles were boiled in 5× loding buffer: 0.25 mol/l Tris-HCl, pH 6.8, 10% SDS, 0.5% bromophenol blue, 5% β-mercaptoethanol, 50% (v/v) glycerol. Western blotting experiments were performed in biologic replicates. Taking the same method, we use rabbit anti-gfp-Pax6-variant antibody to replace rabbit anti-gfp-Pax6 antibody. Experiments were also performed in biologic replicates.

## Results

The *Pax6* gene of *Bufo raddei* Strauch has 3 functional domains. The homology of ORF region is 83% in *Xenopus laevis*. In humans and mice, the homology is 84% and 82%, respectively. The homology of Pax6 protein is greater than 95% in vertebrates. The homology of the paired box is 98%, the paired homeobox is 100%, and PST region is 96% in *Xenopus laevis*.

**Figure 1:**
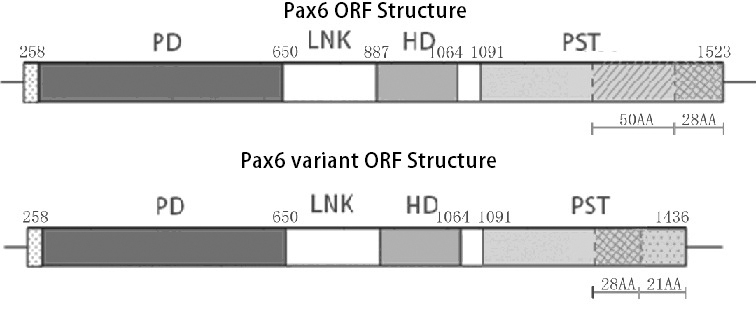
Structural analysis of *Pax6* ORF and *Pax6* variant ORF of *Bufo raddei* Strauch. There is only 53.1% similarity in PST between the *Pax6* and *Pax6* variant in homology analysis employing DNAMAN software (Lynnon Corporation, Quebec, Canada). From the 67th amino acid to the 3 'end, Pax6 variant protein has 49 Amino acids while Pax6 has 78 amino acids.

The ORF region of *Pax6* variant has 1,182 bp. The locus of *Pax6* variant is FJ175151 in Genebank. Compared with *Pax6*, the homolog of ORF nucleotide sequence of *Bufo raddei* Strauch is more than 99% and compared with the *Pax6* variant *B* of *Xenopus laevis*, the homolog is 84%. The analysis based on DNAMAN software (Lynnon Corporation, Quebec, Canada) indicates the *Pax6* variant also has 3 functional domains: the paired box, the paired homeobox, and the PST region. The *Pax6* variant protein has 393 amino acids, among which only the PST region differs from Pax6 of *Bufo raddei* Strauch. Compared with the PST region of Pax6, Pax6 variant protein loses 50 amino acids in 345–394 AA, and because of frame shift, the 395–422 AA differ from the same region of the Pax6 protein, and in the end of the PST region, the Pax6 variant adds 21 amino acids (Figure 1).

**Figure 2:**
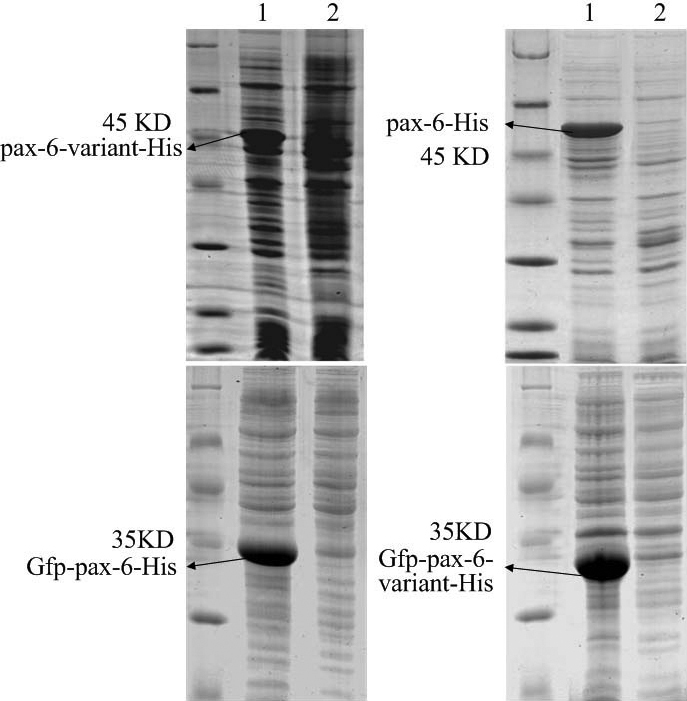
SDS–PAGE for induced expression of recombinant proteins. The 4 recombinant proteins are Pax6-variant-His, Pax6-His, Gfp-Pax6-His, and Gfp-Pax6-variant-His, which were expressed in *E. coli* Rosetta (DE3). Their respective molecular weighs are 43 kDa, 46 kDa, 35 kDa, 32 kDa. All of sample 1 consists of induced proteins; all of sample 2 is negative controls, which were not induced.

*Pax6* and *Pax6* variant of *Bufo raddei* Strauch were cloned and recombinant proteins were expressed successfully in Rosetta (DE3). By SDS–PAGE, 46 kDa and 43 kDa protein bands were observed (Figure 2). His-tagged fusion proteins were purified by a nickel affinity column. At the same time, Gfp-Pax6 and Gfp-Pax6-variant protein were expressed (Figure 2) and purified, and they were used as antigens and injected into the rabbit.

### Yeast two-hybrid system

**Figure 3:**
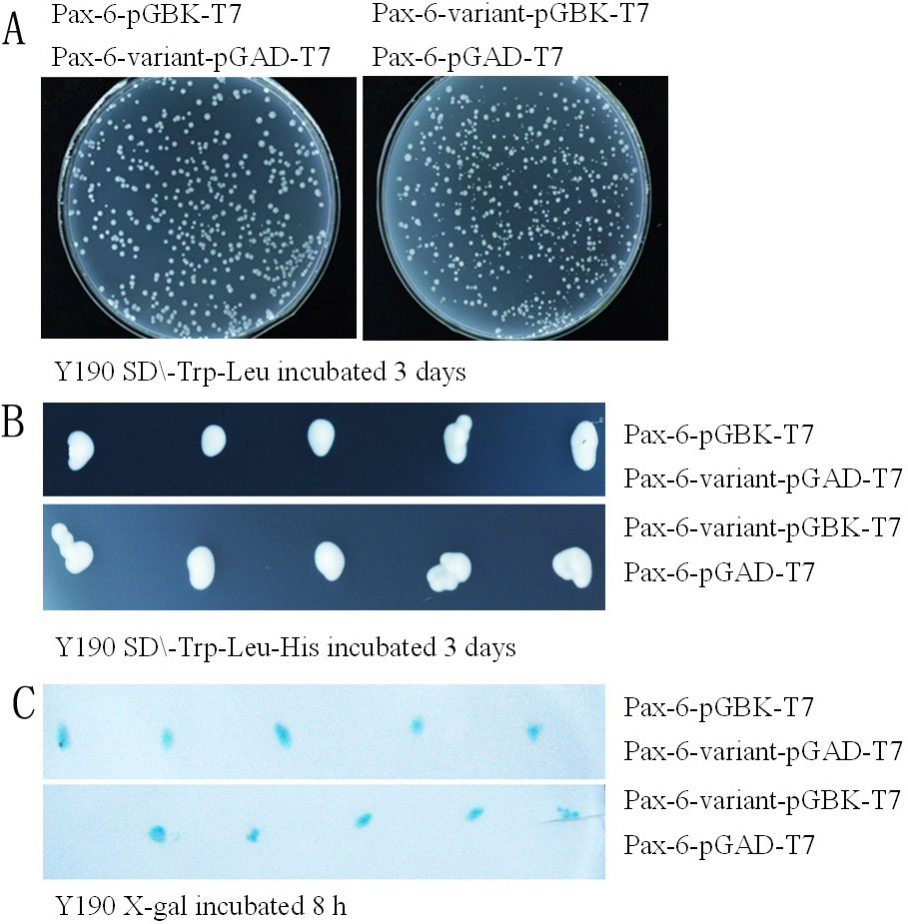
Yeast 2-hybrid system. **A**: Yeast Y190 grow in culture medium without Leu and Trp for 3 days at 30 degrees centigrade; Pax6-pGBK-T7 and Pax6-variant-pGAD-T7 were transformed into the first yeast colony, Pax6-pGAD-T7 and Pax6-variant-pGBK-T7 were transformed into the second yeast colony. **B**: Yeast colony of the experimental group grew in culture medium containing dicyandiamide, without Leu, Trp, and His, after growing for 3 days at 30 °C. **C**: yeast colony transformed into nitrocellulose filter membrane; adding buffer containing x-gal, the color is displayed blue.

Six different Yeast expression systems were produced successfully. When they grew in culture medium lacking Leu, Trp and His for 3 days, only 2 groups could grow, they are Pax6-pGAD-T7 and Pax6-variant-pGBK-T7, and Pax6-pGBK-T7 and Pax6-variant-pGAD-T7. Each group was transferred to nitrocellulose filter membrane, after incubation in x-gal buffer for 8 h at 30 °C, the color of the yeast colony turned blue (Figure 3). This experiment shows only Pax6 and Pax6 variant proteins express in the same cell of yeast y190, the cell activates the reporter gene, and histidine gene 3 (*HIS3*) and β-galactosidase (*LacZ*) were expressed, so the yeast colony could grow in a culture medium that lacked His, and the color of the colony turned blue when incubated in x-gal buffer. The results show that Pax6 and Pax6-variant interact in yeast y190.

### Co-immunoprecipitation

**Figure 4:**
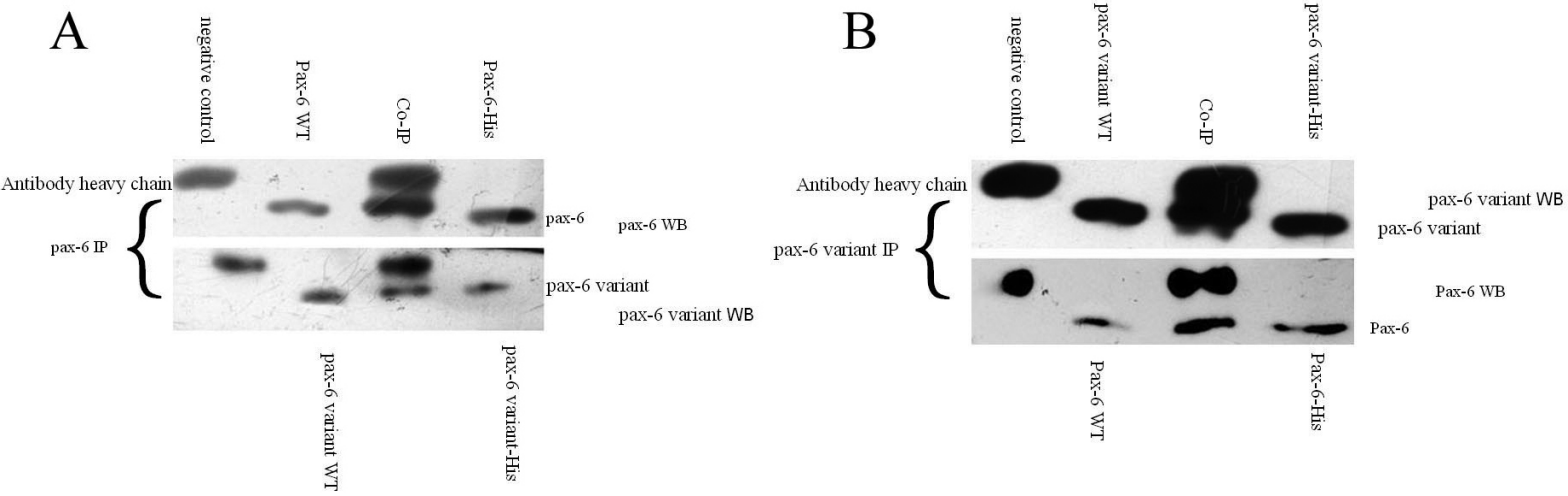
Co-immunoprecipitation of Pax6 and Pax6-variant. **A**: Rabbit anti-Gfp-Pax6 antibody was used to do the IP experiment, and rabbit anti-Gfp-Pax6 antibody was used to do the western blotting. The inputs from left to right are negative control, 6-day embryonic tissue extract, Co-IP, and Pax6-his protein. Pax6 protein is obvious in the 6-day embryonic tissue extract and the Co-IP of the input, and lower than the heavy chain of the antibody. When using the rabbit anti-Gfp-Pax6-varian antibody to do the western blotting, the input from left to right is negative control, 6-day embryonic tissue extract, Co-IP, and Pax6-varian-his protein. Pax6-varian can be found in the 6-day embryonic tissue extract and the Co-IP of the input. **B**: The anti-Gfp-Pax6-varian antibody was used to do the IP experiment. When using anti-Gfp-Pax6-varian antibody and corresponding input, Pax6 variant was probed in the 6-day embryonic tissue extract and the Co-IP of the input; also, Pax6 was probed with an anti-Gfp-Pax6 antibody in the 6-day embryonic tissue extract and the Co-IP of the input. The results show that both Pax6 and Pax6 variant protein can be found in embryonic tissues of the *Bufo raddei* Strauch, and a complex of these proteins was found in the Co-IP of the input. WB: Western Blotting; WT: Wild Type.

First, the rabbit anti-Gfp-Pax6 antibody was used to do immunoprecipitation, rabbit anti-GST antibody was used in negative control, then rabbit anti-Gfp-Pax6 antibody was used to do western blotting. The results show that a heavy chain of antibodies was found in the negative group and experimental group (Co-IP), and Pax6 protein was found in the experimental group and 2 positive controls. When using Gfp-Pax6-variant antibody to do western blotting, the Pax6-His was replaced with Pax6 variant-His in the inputs. The results show that Pax6-variant protein was found in the experiment and 2 positive controls. When using rabbit anti-Gfp-Pax6-variant antibody to do immunoprecipitation, first using anti-Gfp-Pax6-variant antibody to do western blotting, Pax6-variant was found in both the experiment and 2 positive controls. Then using anti-Gfp-Pax6 antibody to do western blotting, Pax6 variant-His was replaced by Pax6-His of the inputs; Pax6 protein was found in both the experiment and 2 positive controls (Figure 4). The experimental group shows that Pax6 and Pax6 variant can form a complex, through rabbit anti-Gfp-Pax6 antibody or rabbit anti-Gfp-Pax6-variant antibody, combined with magnetic Protein A particles. These results suggest that Pax6 and Pax6-variant of *Bufo raddei* Strauch interact in vivo.

## Discussion

The adult *Bufo raddei* Strauch lives mostly on land and feeds on small invertebrates such as insects. Their feeding habits change after they undergo complete metamorphosis, which indicates a kind of adaption to natural selection during its long evolution; thus, they may be higher than *Xenopus* in their evolutionary position. We first found a new *Pax6* mutant of normal *Bufo raddei* Strauch. The *Pax6* mutant may be caused by evolution. Several researchers found that most mutants of *Pax6* were lost in 3′ sequences [44]. The mutant proteins combine with downstream genes that differ from Pax6 [45,46]. The PST region of mutants may contain a part of transcription activation [40]. Some mutations have been found to occur in the COOH-terminal of Pax6, which contains the DNA-binding domains but has lost most of the transactivation domain. These mutants are dominant-negative in transient transfection assays when they are co-expressed with wild-type Pax6 [47]. A mutant of Pax6 was found in normal *Bufo raddei* Strauch; it is located in the PST region of Pax6. It is interesting that it can exist stably in normal *Bufo raddei* Strauch, which differs from previous reports that some mutants can lead to developmental problems in the eyes.

This study found that Pax6 and Pax6 variant were able to interact in yeast, and the co-immunoprecipitation experiment tells us that they still have a mutual effect in *Bufo raddei* Strauch. The results indicate that Pax6 variant and Pax6 protein can form a compound, and can have influence on the function of Pax6. Because the gst-Pax6-variant and gst-Pax6 proteins were not expressed and purified, whether the interactions were direct or indirect is not known from the pull-down experiment, so it is possible that some other proteins are involved in the interactions. What is most significant is that the mutant protein can regulate *Pax6*.

*Pax6* is a key regulator in eye development; it expresses early in cells fated to form the eye and parts of the forebrain, hindbrain, and spinal cord [48]. As an early marker of corneal epithelial cell differentiation, *Pax6* has high-level expression in early cell cultures, but then is found at very low levels in proliferating cells [49]. Thus, we infer that Pax6 is also reduced in *Bufo raddei* Strauch eye development. The combination of Pax6 and pax6 variant protein may result in the downregulation of *Pax6* by their interaction.

Some elements of transcriptional activation involve leucine, proline, glutamic acid, serine, and threonine [50]. We found in the PST region of Pax6 that the ratio of proline, serine, and threonine were 15%, 19%, and 12%, respectively, but in the Pax6 variant, the ratios were 10%, 17%, and 11%, respectively. Using 3D-PSSM software, we found that Pax6 had 2 more α-helixes than the Pax6 variant protein in the PST region, which makes it possible that the Pax6 and Pax6 variant have mutual effects and form a complex structure.

Mutations in *Pax6* lead to a variety of ocular anomalies in human beings and mice. However in *Bufo raddei* Strauch, the *Pax6* variant has no impact on pathological changes, but may have a role in eye development. The mutual effect between *Pax6* and *Pax6* variant may be important for eye development; we assume it to be a special evolution in *Bufo raddei* Strauch. *Pax6* is still pivotal for eye development, but the expression of *Pax6* variant appears to be a necessity.
